# Dapagliflozin decreases small dense low-density lipoprotein-cholesterol and increases high-density lipoprotein 2-cholesterol in patients with type 2 diabetes: comparison with sitagliptin

**DOI:** 10.1186/s12933-016-0491-5

**Published:** 2017-01-13

**Authors:** Toshiyuki Hayashi, Tomoyasu Fukui, Noriko Nakanishi, Saki Yamamoto, Masako Tomoyasu, Anna Osamura, Makoto Ohara, Takeshi Yamamoto, Yasuki Ito, Tsutomu Hirano

**Affiliations:** 1 Division of Diabetes, Metabolism, and Endocrinology, Department of Medicine, Showa University School of Medicine, 1-5-8, Hatanodai, Shinagawa-ku, Tokyo, 142-8666 Japan; 2 Reagent R&D department, Denka Seiken Co., Ltd., Nihonbashi Mitsui Tower, 1-1, Nihonbashi-Muromachi 2-chome, Chuo-ku, Tokyo, 103-8338 Japan

**Keywords:** Dapagliflozin, Sodium-glucose co-transporter-2 inhibitor, Small dense low-density lipoprotein-cholesterol, High-density lipoprotein 2-cholesterol, Lipoprotein subspecies

## Abstract

**Background:**

The sodium-glucose co-transporter-2 (SGLT-2) inhibitors have been reported to increase both low-density lipoprotein (LDL) and high-density lipoprotein (HDL)-cholesterol (C). This study aimed to determine how SGLT-2 inhibitors affect LDL and HDL-C subspecies.

**Methods:**

This single center, open-label, randomized, prospective study included 80 patients with type 2 diabetes taking prescribed oral hypoglycemic agents. Patients were allocated to receive dapagliflozin (n = 40) or sitagliptin (n = 40) as add-on treatment. Fasting blood samples were collected before and 12 weeks after this intervention. Small dense (sd) LDL-C, large buoyant (lb) LDL-C, HDL2-C, and HDL3-C levels were determined using our established homogeneous assays. Statistical comparison of blood parameters before and after treatment was performed using the paired *t* test.

**Results:**

Dapagliflozin and sitagliptin comparably decreased HbA1c (0.75 and 0.63%, respectively). Dapagliflozin significantly decreased body weight, systolic blood pressure, plasma triglycerides and liver transaminases, and increased adiponectin; sitagliptin did not alter these measurements. LDL-C and apolipoprotein (apo) B were not significantly changed by dapagliflozin, whereas HDL-C and apo AI were increased. Dapagliflozin did not alter concentrations of LDL-C, but sd LDL-C decreased by 20% and lb LDL-C increased by 18%. Marked elevation in lb LDL-C (53%) was observed in individuals (n = 20) whose LDL-C was elevated by dapagliflozin. However, sd LDL-C remained suppressed (20%). Dapagliflozin increased HDL2-C by 18% without affecting HDL3-C. Sitagliptin did not alter plasma lipids or lipoprotein subspecies.

**Conclusions:**

A SGLT-2 inhibitor, dapagliflozin suppresses potent atherogenic sd LDL-C and increased HDL2-C, a favorable cardiometabolic marker. Although LDL-C levels are elevated by treatment with dapagliflozin, this was due to increased concentrations of the less atherogenic lb LDL-C. However, these findings were not observed after treatment with dipeptidyl peptidase-4 inhibitor, sitagliptin.

*Trial registration* UMIN Clinical Trials Registry (UMIN000020984)

**Electronic supplementary material:**

The online version of this article (doi:10.1186/s12933-016-0491-5) contains supplementary material, which is available to authorized users.

## Background

Recently, sodium-glucose co-transporter-2 (SGLT-2) inhibitors have been developed as novel therapeutic agents for the treatment of type 2 diabetes [1–3]. These drugs inhibit the reabsorption of glucose in the proximal tubules of the kidney, leading to increased urinary excretion of glucose and decreased levels of blood glucose in diabetic patients [1–3]. In addition, use of SGLT-2 inhibitors results in decreased body weight and visceral fat mass, and reduced blood pressure. These factors are expected to suppress adverse cardiovascular outcomes [1–3]. In a secondary prevention study (the EMPA-REG OUTCOME^®^ trial), empagliflozin successfully suppressed composite adverse cardiovascular outcomes [4]. However, it remains unclear whether SGLT-2 inhibitors prevent cardiovascular events in cardiovascular disease-free diabetic patients. Several clinical studies have revealed that SGLT-2 inhibitors decrease plasma triglyceride (TG) levels and increase high-density lipoprotein (HDL)-cholesterol (C) (HDL-C) levels, but also increase low-density lipoprotein (LDL)-C (LDL-C) levels [5, 6]. LDL-C is the most powerful determinant of cardiovascular events in patients with type 2 diabetes [7, 8], thus, the increased levels of LDL-C associated with SGLT-2 inhibitor use might increase cardiovascular risk.

LDL-C can be fractionated into large buoyant (lb) and small dense (sd) particles based on size and density. An abundance of clinical evidence has shown that sd LDL-C particles are more atherogenic than lb LDL-C particles, and that the predominance of sd LDL-C confers a threefold increased risk for coronary artery disease [9], as sd LDL-C forms a good substrate for oxidized LDL-C in the arterial wall [10]. LDL size is inversely regulated by plasma TG levels [11] and insulin resistance [12]. There is a preponderance of sd LDL-C particles in individuals with hypertriglyceridemia, metabolic syndrome [13], and type 2 diabetes [14].

We established a homogeneous assay for the direct measurement of serum sd LDL-C concentrations [15]. This assay has been used in well-known, large cohort studies which revealed that sd LDL-C concentrations more sensitively predicted cardiovascular events than did LDL-C or lb LDL-C concentrations [16–18]. We hypothesized that SGLT-2 inhibitors decrease the levels of potent atherogenic sd LDL-C particles by decreasing TG levels and enhancing insulin sensitivity [1, 2]. If so, an increase in LDL-C by SGLT-2 inhibitors may be attributable to increases in less atherogenic lb LDL-C particles. Hence, we investigated the effect on plasma concentrations of LDL-C subspecies in type 2 diabetic patients treated with the SGLT-2 inhibitor, dapagliflozin, compared with patients treated with the dipeptidyl peptidase-4 (DPP-4) inhibitor, sitagliptin.

HDL-C also has subspecies, namely HDL2-C and HDL3-C. Large, cholesterol-rich HDL2-C is inversely associated with plasma TG and insulin resistance, whereas small, cholesterol-poor HDL3-C is not [19]. We established a homogeneous assay for the direct measurement of HDL3-C serum concentrations. Subtracting the concentration of HDL3-C from HDL-C gives the serum concentration of HDL2-C [20]. The second aim of the present study was to determine how the use of SGLT-2 inhibitor affects HDL-C subspecies in patients with type 2 diabetes.

## Methods

### Study design and subjects

This study was a single center, open-label, randomized, prospective study. Dapagliflozin (5 mg) or sitagliptin (50 mg) was administered once daily for 12 weeks as add-on therapy to established oral hypoglycemic drug treatment (sulfonylureas, metformin, or an α-glucosidase inhibitor). The study was conducted in patients diagnosed with type 2 diabetes whose blood glucose was inadequately controlled despite combined treatment with diet/exercise and an oral hypoglycemic drug for more than 12 weeks prior to screening. The main inclusion criteria were: (1) age ≥20 years and ≤65 years, (2) diagnosis of type 2 diabetes at least 3 months prior to screening, and (3) HbA1c of ≥6.5% [48 mmol/mol] and ≤9.4% [79 mmol/mol] whilst on treatment. The main exclusion criteria were: (1) previous stroke or ischemic heart disease, (2) insulin use, (3) current or potential pregnancy, (4) an estimated glomerular filtration rate of <60 mL/min/1.73 m^2^ at the beginning of the run-in period, (5) a TG level of ≥600 mg/dL on the day of screening, and (6) users of omega-3 fatty acids. No drugs were changed during the study period.

### Measurements

Overnight fasting blood samples were obtained before and 12 weeks after the administration of dapagliflozin or sitagliptin. LDL-C and HDL-C were measured by conventional direct methods. Both sd LDL-C and HDL3-C concentrations were measured using the homogeneous methods we established [15, 20]. Non HDL-C was estimated by subtracting the HDL-C from the total-C concentration. Concentrations of lb LDL-C and HDL2-C were estimated by subtracting the sd LDL-C from the LDL-C or subtracting the HDL3-C from the HDL-C, respectively [15, 20]. We have previously reported excellent correlations (coefficient of correlation: r > 0.90, p < 0.0001) between measurements obtained using our homogeneous and the standard ultracentrifugation methods of measuring LDL-C and HDL-C sub-fraction concentrations [15, 20]. In addition, the validity of our homogeneous methods has been evaluated by other researchers [21]. Remnant-like particle-cholesterol (RLP-C) was measured by the direct homogeneous method (MetaboRead, Kyowa Medex Co., Ltd., Tokyo, Japan). Total adiponectin was determined by immunoassay (Denka Seiken., Co., Ltd., Tokyo, Japan).

### Statistical analysis

Data were expressed as mean ± standard deviation (SD), number and percentage, or percent changes after treatment. Statistical analyses were performed using JMP 11.0 (SAS Institute., Cavy, NC, USA). The unpaired t-test (for continuous variables) or Fisher’s exact test (for categorical variables) was used for statistical analysis of differences in the baseline clinical parameters of participants in the dapagliflozin and sitagliptin groups. Comparison of plasma parameters before and after treatment was performed using the paired t-test, and for intergroup comparisons, the unpaired t-test was used for normally distributed data, the Mann–Whitney U test for data with skewed distributions. Correlations between 2 variables, the Pearson correlation coefficient was used for data with normal distribution pattern, whereas the Spearman rank-correlation coefficient was used for data with a non-normal distribution. Differences were considered statistically significant at values of p < 0.05.

## Results

The study included 80 participants, 62 men and 18 women, who were randomly allocated to receive dapagliflozin (n = 40) or sitagliptin (n = 40). Additional file 1: Table S1 lists baseline general characteristics and blood biochemical measurements, Additional file 1: Table S2 lists blood lipid levels. There were no significant differences in general characteristics, blood biochemistry results, or lipid profiles between the dapagliflozin and sitagliptin group at baseline. Both groups exhibited hyperglycemia, mild obesity (BMI = 28 kg/m^2^), and mild liver dysfunction. The majority of patients had normal total cholesterol, LDL-C, and HDL-C levels and mild hypertriglyceridemia.

Table 1 lists general characteristics and blood biochemical measurements and lipid levels at 12 weeks after treatment with dapagliflozin or sitagliptin, and how these changed from baseline measurements. Dapagliflozin significantly reduced body weight (by 2.2 kg) (p < 0.001) and systolic blood pressure (by 4 mmHg) (p = 0.022), whereas these changes were not observed with sitagliptin. HbA1c levels decreased 0.75% in the dapagliflozin group vs. 0.63% in the sitagliptin group. Fasting blood glucose levels decreased by 23.5 and 18.7 mg/dL in the dapagliflozin and sitagliptin groups, respectively. These results are consistent with the results reported in previous clinical trials. Aspartate aminotransferase (AST) and alanine aminotransferase (ALT) were significantly (p < 0.001) decreased by dapagliflozin, while the liver function remained unchanged by sitagliptin treatment. Hemoglobin, hematocrit and blood urea nitrogen were significantly increased in the dapagliflozin group (p < 0.001), while this remained unchanged in the sitagliptin group. Dapagliflozin significantly increased the plasma level of adiponectin from 6.0 ± 3.4 to 7.6 ± 4.2 ng/mL (p < 0.001), whereas sitagliptin had no effect on plasma adiponectin levels. Thus, there were significantly differences between two treatment groups in terms of changes in ALT, AST, hemoglobin, hematocrit and adiponectin (p < 0.01).

**Table 1:** Clinical parameters before and after administration of dapagliflozin or sitagliptin

	Dapagliflozin (n = 40)				Sitagliptin (n = 40)				p value^b^
	Pre treatment	Post treatment	% change	p value^a^	Pre treatment	Post treatment	% change	p value^a^	
BW (kg)	78.4 ± 14.3	76.2 ± 1.8	−2.8	<0.001*	77.6 ± 11.6	77.7 ± 11.6	0.1	0.089	0.042*
SBP (mmHg)	130.7 ± 15.8	126.5 ± 12.7	−3.2	0.022*	133.2 ± 17.8	131.0 ± 13.3	−1.7	0.611	0.031*
DBP (mmHg)	86.9 ± 10.7	82.3 ± 16.0	−5.3	0.188	88.1 ± 9.5	84.4 ± 11.0	−4.2	0.024*	0.242
HR (bpm)	82.5 ± 10.4	81.6 ± 13.9	−1.1	0.300	81.5 ± 10.3	77.1 ± 10.8	−5.4	0.056	0.344
Hb (mg/dL)	14.4 ± 1.2	15.1 ± 1.4	4.9	<0.001*	14.2. ± 1.4	14.5 ± 1.5	2.1	0.088	<0.001*
Ht (%)	41.9 ± 3.5	44.5 ± 4.0	6.2	<0.001*	41.4 ± 4.3	41.9 ± 4.2	1.2	0.297	<0.001*
AST (IU/L)	34.5 ± 19.4	26.8 ± 12.8	−22.3	<0.001*	33.2 ± 9.8	35.4 ± 14.9	6.6	0.089	<0.001*
ALT (IU/L)	46.6 ± 37.0	33.5 ± 24.9	−28.1	<0.001*	42.8 ± 15.0	44.9 ± 18.4	4.9	0.202	<0.001*
γGTP (IU/L)	53.2 ± 43.0	42.3 ± 47.2	−20.5	0.109	50.9 ± 18.1	52.2 ± 22.0	2.6	0.729	0.107
BUN (mg/dL)	14.6 ± 4.3	16.8 ± 5.0	15.1	<0.001*	13.5 ± 4.5	15.3 ± 4.6	13.3	0.115	0.367
Cre (mg/dL)	0.72 ± 0.17	0.74 ± 0.23	2.8	0.173	0.77 ± 0.17	0.81 ± 0.18	5.2	0.110	0.757
eGFR (mL/min/1.73 m^2^)	86.2 ± 18.4	83.6 ± 23.2	−3.0	0.230	83.5 ± 22.7	79.2 ± 21.4	−5.1	0.003*	0.545
FPG (mg/dL)	145.8 ± 47.8	122.3 ± 24.9	−16.1	0.002*	144.9 ± 57.9	126.2 ± 43.9	−12.9	0.043*	0.673
HbA1c (%)	7.61 ± 1.15	6.86 ± 0.81	−9.9	<0.001*	7.55 ± 1.64	6.92 ± 1.20	−8.3	0.006*	0.378
C-peptide (ng/mL)	2.79 ± 1.39	2.40 ± 1.56	−14.0	0.018*	2.70 ± 1.25	2.78 ± 1.48	3.0	0.633	0.190
Adiponectin (ng/mL)	6.0 ± 3.4	7.6 ± 4.2	26.7	<0.001*	6.2 ± 5.3	6.2 ± 3.8	0	0.899	0.002*

Data are expressed as mean ± standard deviation or percent changes after the treatment

*BW* body weight, *SBP* systolic blood pressure, *DBP* diastolic blood pressure, *HR* heart rate, *Hb* hemoglobin, *Ht* hematocrit, *AST* aspartate aminotransferase, *ALT* alanine aminotransferase, *γGTP* γ-glutamyltranspeptidase, *BUN* blood urea nitrogen, *Cre* creatinine, *eGFR* estimated glomerular filtration rate, *FPG* fasting plasma glucose

^a^p values for the intragroup comparison (pre vs. post treatment values in dapagliflozin or sitagliptin group, * p < 0.05)

^b^p values for intergroup comparison (dapagliflozin vs. sitagliptin group in the changes from pre to post treatment, * p < 0.05)

**Fig. 1:**
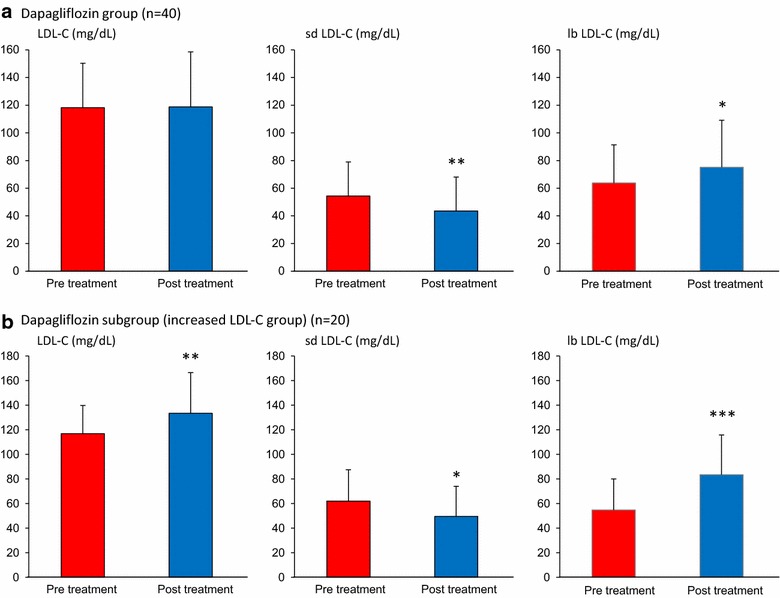
Effects of dapagliflozin on LDL-C and its subspecies. Data are expressed as mean ± standard deviation. LDL-C and its subspecies values in the dapagliflozin group (**a**) or subgroup whose LDL-C was increased by dapagliflozin treatment (**b**) were compared between before and after the treatment. *p < 0.05, **p < 0.01, ***p < 0.001 (pre vs. post treatment values). *LDL-C* low-density lipoprotein-cholesterol, *sd LDL-C* small dense LDL-cholesterol, *lb LDL-C* large buoyant LDL-cholesterol

**Fig. 2:**
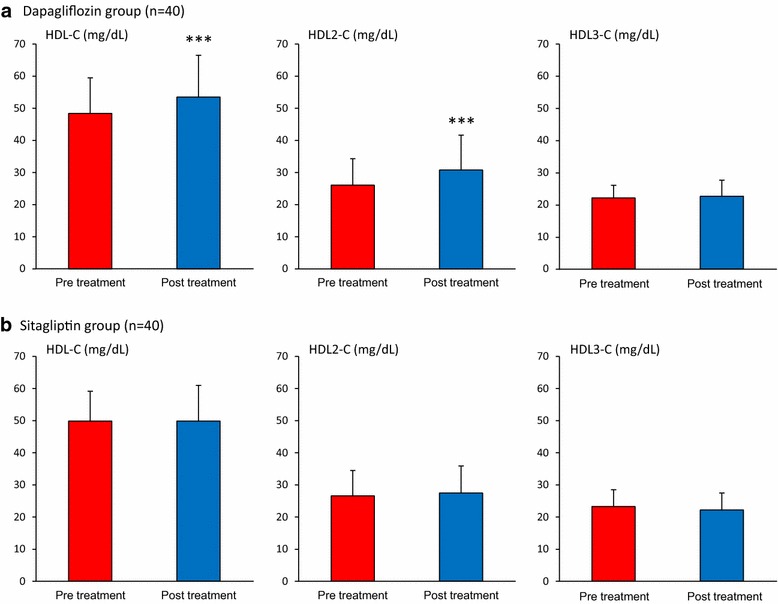
Effects of dapagliflozin and sitagliptin on HDL-C and its subspecies. Data are expressed as mean ± standard deviation. HDL-C and its subspecies values in the dapagliflozin group (**a**) or sitagliptin group (**b**) were compared between before and after the treatment. ***p < 0.001 (pre vs. post treatment values). *HDL-C* high-density lipoprotein-cholesterol, *HDL2-C* high-density lipoprotein 2-cholesterol, *HDL3-C* high-density lipoprotein 3-cholesterol

Total-C, LDL-C, and apolipoprotein (apo) B were unchanged in both groups (Table 2). In the dapagliflozin group, the concentration of sd LDL-C decreased significantly (20%, p < 0.01), whereas that of lb LDL-C increased significantly (18%, p < 0.05) (Fig. 1a). These changes were not observed in sitagliptin group. HDL-C, HDL2-C, apo AI, apo AII were significantly increased in dapagliflozin group (p < 0.05) (Fig. 2a); these changes were not observed in sitagliptin group (Fig. 2b). Thus, there were significantly differences between two treatment groups in terms of changes in sd LDL-C, lb LDL-C, HDL-C, HDL2-C and apo AI (Table 2) (p < 0.05).

**Table 2:** Lipid parameters before and after administration of dapagliflozin or sitagliptin

	Dapagliflozin (n = 40)				Sitagliptin (n = 40)				p value^b^
	Pre treatment	Post treatment	% change	p value^a^	Pre treatment	Post treatment	% change	p value^a^	
Total-C (mg/dL)	193.5 ± 36.6	198.4 ± 45.9	2.5	0.863	192.5 ± 58.2	195.5 ± 38.9	1.6	0.720	0.102
TG (mg/dL)	152.6 ± 63.7	133.7 ± 75.8	−12.4	0.145	150.2 ± 85.2	144.6 ± 87.2	−3.7	0.245	0.928
HDL-C (mg/dL)	48.4 ± 11.1	53.5 ± 13.0	10.5	<0.001*	50.3 ± 9.25	50.3 ± 11.1	0	0.948	0.003*
LDL-C (mg/dL)	118.2 ± 32.1	118.8 ± 39.7	0.5	0.875	120.2 ± 35.1	114.7 ± 33.1	−4.6	0.257	0.323
Non HDL-C (mg/dL)	145.1 ± 36.0	144.9 ± 439	−0.1	0.947	142.2 ± 57.3	145.1 ± 38.4	2.9	0.717	0.328
Apo AI (mg/dL)	133.5 ± 21.6	143.5 ± 22.6	7.5	0.002*	134.9 ± 25.2	128.8 ± 30.7	−4.5	0.135	0.002*
Apo AII (mg/dL)	29.7 ± 4.5	30.9 ± 5.0	4.0	0.022*	30.5 ± 7.2	30.5 ± 7.2	0	0.945	0.148
Apo B (mg/dL)	100.1 ± 24.5	100.8 ± 28.1	0.7	0.777	102.3 ± 27.7	97.4 ± 26.5	−4.8	0.123	0.131
Apo CII (mg/dL)	4.8 ± 1.7	4.9 ± 2.2	2.1	0.362	5.4 ± 2.8	5.1 ± 2.6	−5.6	0.197	0.105
Apo CIII (mg/dL)	10.5 ± 3.2	11.4 ± 4.3	8.6	0.021*	10.1 ± 4.5	9.6 ± 4.0	−5.0	0.335	0.028*
Apo E (mg/dL)	4.4 ± 1.2	4.4 ± 1.5	0	0.530	4.3 ± 1.5	4.1 ± 1.0	−4.7	0.296	0.216
RLP-C (mg/dL)	6.8 ± 4.3	6.4 ± 5.1	−5.9	0.620	6.9 ± 8.0	6.1 ± 5.7	−11.6	0.303	0.750
HDL2-C (mg/dL)	26.1 ± 8.2	30.8 ± 10.8	18.0	<0.001*	26.6 ± 7.9	27.5 ± 8.4	3.4	0.334	0.013*
HDL3-C (mg/dL)	22.2 ± 3.9	22.7 ± 5.0	2.3	0.527	23.3 ± 5.2	22.2 ± 5.3	−4.7	0.132	0.130
sd LDL-C (mg/dL)	54.4 ± 24.6	43.6 ± 24.4	−19.9	0.005*	54.0 ± 22.5	50.4 ± 22.4	−6.7	0.368	0.003*
lb LDL-C (mg/dL)	63.8 ± 27.6	75.1 ± 34.1	17.7	0.026*	66.2 ± 26.3	64.3 ± 24.1	−2.9	0.671	0.029*

Data are expressed as mean ± standard deviation or percent changes after the treatment

*Total-C* total-cholesterol, *TG* triglycerides, *HDL-C* high-density lipoprotein-cholesterol, *LDL-C* low-density lipoprotein-cholesterol, *Apo* apolipoprotein, *RLP-C* remant-like particles-cholesterol, *sd LDL-C* small dense LDL-cholesterol, *lb LDL-C* large buoyant LDL-cholesterol, *HDL2-C* high-density lipoprotein 2-cholesterol, *HDL3-C* high-density lipoprotein 3-cholesterol

^a^p values for the intragroup comparison (pre vs. post treatment values in dapagliflozin or sitagliptin group, * p < 0.05)

^b^p values for intergroup comparison (dapagliflozin vs. sitagliptin group in the changes from pre to post treatment, * p < 0.05)

The correlation between the changes in LDL-C subspecies, HDL-C subspecies and changes in clinical and lipid profile after 12 weeks of treatment with either dapagliflozin or sitagliptin was analyzed in Tables 3 and 4, respectively. Dapagliflozin-mediated changes in LDL-C (r = 0.894, p < 0.001) and lb LDL-C (r = 0.665, p < 0.001) correlated with changes in apo B levels, while changes in sd LDL-C was negatively correlated with only changes in lb LDL-C (r = −0.690, p < 0.001). Sitagliptin-mediated changes in LDL-C (r = 0.909, p < 0.001), sd LDL-C (r = 0.467, p < 0.01) and lb LDL-C (r = 0.377, p < 0.05) were correlated with changes in apo B levels. Sitagliptin-mediated changes in sd LDL-C were also correlated with changes in apo CIII level (r = 0.451, p < 0.01).

**Table 3:** Correlation between the changes of LDL-C subspecies, HDL-C subspecies and the changes of clinical parameters

	Dapagliflozin						Sitagliptin					
	ΔLDL-C (mg/dL)		Δsd LDL-C (mg/dL)		Δlb LDL-C (mg/dL)		ΔLDL-C (mg/dL)		Δsd LDL-C (mg/dL)		Δlb LDL-C (mg/dL)	
	r	p	r	p	r	p	r	p	r	p	r	p
ΔBW (kg)	0.042	0.795	−0.191	0.241	0.174	0.287	0.088	0.588	−0.084	0.607	0.017	0.917
ΔHbA1c (%)	0.109	0.506	−0.118	0.472	0.168	0.305	0.217	0.190	−0.025	0.088	0.280	0.087
ΔC-peptide (ng/mL)	0.208	0.229	0.130	0.455	0.055	0.750	0.516	0.023*	−0.133	0.586	0.648	0.002*
ΔAST (IU/L)	0.008	0.957	−0.019	0.906	−0.019	0.906	0.188	0.388	0.286	0.195	−0.138	0.527
ΔALT (IU/L)	0.038	0.816	0.167	0.308	−0.097	0.555	0.026	0.906	0.232	0.297	−0.210	0.334
ΔγGTP (IU/L)	0.220	0.176	0.186	0.256	0.020	0.900	0.291	0.177	0.465	0.029*	−0.267	0.217

	ΔHDL-C (mg/dL)		ΔHDL2-C (mg/dL)		ΔHDL3-C (mg/dL)		ΔHDL-C (mg/dL)		ΔHDL2-C (mg/dL)		ΔHDL3-C (mg/dL)	
	r	p	r	p	r	p	r	p	r	p	r	p
ΔBW (kg)	−0.189	0.247	−0.208	0.203	0.002	0.986	0.107	0.509	−0.098	0.545	−0.221	0.169
ΔHbA1c (%)	−0.046	0.780	−0.073	0.656	0.036	0.826	0.263	0.109	0.329	0.043*	0.148	0.378
ΔC-peptide (ng/mL)	−0.074	0.671	−0.165	0.342	0.127	0.463	0.095	0.698	0.255	0.291	−0.300	0.211
ΔAST (IU/L)	0.220	0.178	0.220	0.177	0.029	0.860	−0.394	0.062	−0.347	0.104	0.072	0.742
ΔALT (IU/L)	0.164	0.316	0.168	0.306	0.017	0.915	−0.289	0.180	−0.263	0.225	0.052	0.810
ΔγGTP (IU/L)	0.197	0.227	0.153	0.350	0.095	0.563	0.201	0.570	−0.180	0.395	0.222	0.308

*r* coefficient of correlation, *BW* body weight, *AST* aspartate aminotransferase, *ALT* alanine aminotransferase, *γGTP* γ-glutamyltranspeptidase, *LDL-C* low-density lipoprotein-cholesterol, *sd LDL-C* small dense LDL-cholesterol, *lb LDL-C* large buoyant LDL-cholesterol, *HDL-C* high-density lipoprotein-cholesterol, *HDL2-C* high-density lipoprotein 2-cholesterol, *HDL3-C* high-density lipoprotein 3-cholesterol

* p < 0.05

**Table 4:** Correlation between the changes of LDL-C subspecies, HDL-C subspecies and the changes of lipid parameters

	Dapagliflozin						Sitagliptin					
	ΔLDL-C (mg/dL)		Δsd LDL-C (mg/dL)		Δlb LDL-C (mg/dL)		ΔLDL-C (mg/dL)		Δsd LDL-C (mg/dL)		Δlb LDL-C (mg/dL)	
	r	p	r	p	r	p	r	p	r	p	r	p
ΔTG (mg/dL)	0.085	0.604	0.008	0.959	0.055	0.736	0.125	0.430	0.155	0.345	−0.009	0.953
ΔLDL-C (mg/dL)			0.080	0.625	0.665	<0.001*			0.425	0.006*	0.507	0.001*
Δsd LDL-C (mg/dL)	0.080	0.625			−0.690	<0.001*	0.425	0.006*			−0.324	0.043*
Δlb LDL-C (mg/dL)	0.665	<0.001*	−0.690	<0.001*			0.507	0.001*	−0.324	0.043*		
Δapo AI (mg/dL)	0.320	0.046*	0.280	0.084	0.022	0.890	0.301	0.073	0.044	0.795	0.013	0.938
Δapo AII (mg/dL)	0.466	0.002*	0.270	0.095	0.135	0.410	0.584	<0.001*	0.241	0.150	0.198	0.238
Δapo B (mg/dL)	0.894	<0.001*	0.078	0.639	0.592	<0.001*	0.909	<0.001*	0.467	0.003*	0.377	0.021*
Δapo CIII (mg/dL)	0.316	0.049	0.247	0.128	0.044	0.787	0.287	0.084	0.451	0.005*	−0.135	0.422

	ΔHDL-C (mg/dL)		ΔHDL2-C (mg/dL)		ΔHDL3-C (mg/dL)		ΔHDL-C (mg/dL)		ΔHDL2-C (mg/dL)		ΔHDL3-C (mg/dL)	
	r	p	r	p	r	p	r	p	r	p	r	p
ΔTG (mg/dL)	−0.156	0.341	−0.125	0.446	−0.069	0.674	−0.176	0.277	−0.074	0.646	−0.228	0.155
ΔLDL-C (mg/dL)	0.295	0.067	0.167	0.306	0.239	0.142	0.555	<0.001*	0.254	0.113	0.307	0.053
Δsd LDL-C (mg/dL)	0.183	0.262	−0.066	0.688	0.411	0.009*	0.192	0.240	−0.036	0.825	0.392	0.013*
Δlb LDL-C (mg/dL)	0.760	0.641	0.171	0.297	−0.134	0.413	0.288	0.071	0.561	<0.001*	0.307	0.053
Δapo AI (mg/dL)	0.890	<0.001*	0.697	<0.001*	0.450	0.004*	0.808	<0.001*	0.516	0.002*	0.489	0.003*
Δapo AII (mg/dL)	0.647	<0.001*	0.394	0.012*	0.500	0.001*	0.633	<0.001*	0.354	0.034*	0.446	0.007*
ΔapoB (mg/dL)	0.283	0.080	0.240	0.141	0.105	0.522	0.368	0.019*	0.135	0.406	0.230	0.153
ΔapoCIII (mg/dL)	0.309	0.055	0.234	0.149	0.156	0.340	0.132	0.415	0.026	0.871	0.100	0.538

*r* coefficient of correlation, *TG* triglycerides, *LDL-C* low-density lipoprotein-cholesterol, *sd LDL-C* small dense LDL-cholesterol, *lb LDL-C* large buoyant LDL-cholesterol, *Apo* apolipoprotein, *HDL-C* high-density lipoprotein-cholesterol, *HDL2-C* high-density lipoprotein 2-cholesterol, *HDL3-C* high-density lipoprotein 3-cholesterol

* p < 0.05

Dapagliflozin-mediated changes in HDL-C and HDL2-C were significantly correlated with changes in apo AI and apo AII levels (p < 0.05). Sitagliptin-mediated changes in HDL-C was correlated with change in LDL-C (r = 0.555, p < 0.001), apo B (r = 0.368, p < 0.05), apo AI (r = 0.808, p < 0.001) and apo AII concentrations (r = 0.633, p < 0.001). Sitagliptin-mediated changes in HDL2-C concentrations were significantly correlated with changes in HbA1c (r = 0.329, p < 0.05) and lb LDL-C (r = 0.561, p < 0.001) (Tables 3 and 4).

Tables 5 and 6 lists the general characteristics and blood lipid levels associated with dapagliflozin use in patients, stratified according to ΔLDL-C (> 0 vs. ≤ 0 mg/dL). In the increased LDL-C subgroup in which LDL-C concentrations increased by 14% (p < 0.01), sd LDL-C decreased significantly (20%, p < 0.05), while lb LDL-C concentrations increased significantly by 53% (p < 0.001) (Fig. 1b). In the decreased LDL-C subgroup in which LDL-C concentrations decreased, both sd LDL-C and lb LDL-C levels decreased significantly by 19 and 10%, respectively (p < 0.05). We compared baseline parameters between the increased LDL-C and the decreased LDL-C groups. In the increased LDL-C group, ages were significantly younger, baseline TG and sd LDL-C levels were significantly higher, and baseline apo AI, HDL2-C and lb LDL-C levels were significantly lower than the decreased LDL-C group (p < 0.05) (Tables 5 and 6).

**Table 5:** Clinical parameters with dapagliflozin in increased LDL-C and decreased LDL-C subgroup

	Increased LDL-C group (n = 20)				Decreased LDL-C group (n = 20)				p values^b^	p values^c^
	Pre treatment	Post treatment	% change	p values^a^	Pre treatment	Post treatment	% change	p values^a^		
Age (years)	51.6 ± 8.2				56.9 ± 7.8				0.047*	
BW (kg)	78.8 ± 14.3	76.8 ± 15.4	−2.5	0.002*	78.0 ± 14.7	75.5 ± 14.5	−3.2	0.001*	0.853	0.394
AST (IU/L)	36.1 ± 22.3	29.4 ± 15.9	−18.6	<0.001*	32.7 ± 16.3	24.1 ± 7.7	−26.3	0.002*	0.596	0.622
ALT (IU/L)	52.5 ± 46.1	39.9 ± 31.9	−24.0	0.032*	40.4 ± 23.6	26.7 ± 11.7	−33.9	0.030*	0.312	0.874
γGTP (IU/L)	51.0 ± 40.5	50.3 ± 62.6	−1.4	0.930	55.5 ± 46.5	34.0 ± 20.4	−38.7	0.030*	0.750	0.119
BUN (mg/dL)	14.1 ± 3.8	16.3 ± 4.3	15.6	0.013*	15.1 ± 4.9	17.3 ± 5.8	14.6	0.010*	0.470	0.996
Cre (mg/dL)	0.71 ± 0.15	0.72 ± 0.17	1.4	0.700	0.72 ± 0.19	0.77 ± 0.29	6.9	0.170	0.964	0.258
eGFR (mL/min/1.73 m^2^)	89.0 ± 18.8	88.0 ± 17.6	−1.1	0.661	83.3 ± 18.1	81.7 ± 22.1	−1.9	0.507	0.342	0.861
FPG (mg/dL)	152 ± 46	127 ± 27	−16.4	0.006*	138 ± 50.1	116 ± 21	−15.9	0.040*	0.382	0.815
HbA1c (%)	7.51 ± 1.1	6.92 ± 0.94	−7.9	0.002*	7.72 ± 1.1	6.81 ± 0.67	−11.8	<0.001*	0.575	0.225
CPR index	2.77 ± 15.2	2.35 ± 1.28	−15.2	0.008*	2.82 ± 1.28	2.44 ± 1.82	−13.4	0.243	0.922	0.486
Adiponectin (ng/mL)	5.5 ± 3.8	7.2 ± 5.4	30.9	0.002*	6.5 ± 2.8	8.0 ± 2.6	23.1	0.001*	0.383	0.675

Data are expressed as mean ± standard deviation or percent changes after the treatment. Clinical and laboratory parameters in the subgroup whose LDL-C was increased or decreased by dapagliflozin treatment were analyzed

*BW* body weight, *AST* aspartate aminotransferase, *ALT* alanine aminotransferase, *γGTP* γ-glutamyltranspeptidase, *BUN* blood urea nitrogen, *Cre* creatinine, *FPG* fasting plasma glucose, *LDL-C* low-density lipoprotein-cholesterol

^a^p values for the intragroup comparison (pre vs. post treatment values in each subgroup, * p < 0.05)

^b^p values for intergroup comparison (increased LDL-C vs. decreased LDL-C group in the pre treatment values, * p < 0.05)

^c^p values for intergroup comparison (increased LDL-C vs. decreased LDL-C group in the changes from pre to post treatment, * p < 0.05)

**Table 6:** Lipid parameters with dapagliflozin in increased LDL-C and decreased LDL-C subgroup

	Increased LDL-C group (n = 20)				Decreased LDL-C group (n = 20)				p values^b^	p values^c^
	Pre treatment	Post treatment	% change	p values^a^	Pre treatment	Post treatment	% change	p values^a^		
Total-C (mg/dL)	188.9 ± 31.3	211.4 ± 43.1	11.9	<0.001*	198.4 ± 41.8	184.8 ± 45.8	−6.9	0.002*	0.428	<0.0001*
TG (mg/dL)	161.3 ± 69.5	151.6 ± 89.4	−6.0	0.440	123.0 ± 51.8	114.8 ± 54.6	−6.7	0.360	0.029*	0.920
HDL-C (mg/dL)	45.4 ± 8.5	52.0 ± 9.7	14.5	0.001*	51.5 ± 12.7	55.1 ± 16.0	7.0	0.070	0.082	0.242
LDL-C (mg/dL)	116.8 ± 23.0	133.4 ± 33.1	14.2	0.002*	119.8 ± 40.1	103.5 ± 41.0	−13.6	<0.001*	0.771	<0.0001*
Apo AI (mg/dL)	126.1 ± 17.8	140.4 ± 18.4	11.3	<0.001*	141.4 ± 22.9	146.8 ± 26.4	3.8	0.230	0.025*	0.145
Apo AII (mg/dL)	29.5 ± 4.8	32.3 ± 5.1	9.5	<0.001*	29.9 ± 4.3	29.5 ± 4.6	−1.3	0.530	0.798	0.001*
Apo B (mg/dL)	100.9 ± 17.6	111.8 ± 22.3	10.8	<0.001*	99.3 ± 30.0	85.0 ± 34.7	−14.4	0.014*	0.846	<0.0001*
Apo CII (mg/dL)	4.8 ± 1.8	5.4 ± 2.5	12.5	0.057	4.7 ± 1.6	4.5 ± 1.8	−4.3	0.270	0.855	0.029*
Apo CIII (mg/dL)	10.7 ± 3.6	12.0 ± 4.8	12.1	0.035*	10.2 ± 2.8	10.7 ± 3.8	4.9	0.340	0.698	0.240
Apo E (mg/dL)	4.3 ± 1.1	4.6 ± 1.7	7.0	0.280	4.4 ± 1.2	4.3 ± 1.3	−2.3	0.420	0.904	0.193
RLP-C (mg/dL)	7.6 ± 5.0	7.8 ± 2.5	2.6	0.870	5.7 ± 3.3	5.0 ± 3.3	−12.3	0.090	0.136	0.809
HDL2-C (mg/dL)	23.5 ± 5.1	29.1 ± 7.8	23.8	<0.001*	28.8 ± 10.03	32.5 ± 13.3	12.8	0.080	0.043*	0.437
HDL3-C (mg/dL)	21.8 ± 4.0	22.8 ± 4.9	4.6	0.410	22.6 ± 3.6	22.5 ± 5.2	−0.4	0.890	0.476	0.450
sd LDL-C (mg/dL)	62.0 ± 25.5	49.5 ± 24.5	−20.2	0.048*	46.3 ± 21.4	37.3 ± 23.2	−19.4	0.015*	0.046*	0.655
lb LDL-C (mg/dL)	54.7 ± 25.3	83.5 ± 32.3	52.7	<0.001*	73.4 ± 27.3	66.1 ± 34.4	−9.9	0.010*	0.033*	<0.0001*
sd LDL-C/LDL-C	0.53 ± 0.18	0.37 ± 0.17	−30.2	0.003*	0.38 ± 0.00	0.35 ± 0.17	−7.9	0.250	0.005*	0.021*

Data are expressed as mean ± standard deviation or percent changes after the treatment. Clinical and laboratory parameters in the subgroup whose LDL-C was increased or decreased by dapagliflozin treatment were analyzed

*Total-C* total-cholesterol, *TG* triglycerides, *HDL-C* high-density lipoprotein-cholesterol, *LDL-C* low-density lipoprotein-cholesterol, *Apo* apolipoprotein, *RLP-C* remant-like particles-cholesterol, *HDL2-C* high-density lipoprotein 2-cholesterol, *HDL3-C* high-density lipoprotein 3-cholesterol, *sd LDL-C* small dense LDL-cholesterol, *lb LDL-C* large buoyant LDL-cholesterol

^a^p values for the intragroup comparison (pre vs. post treatment values in each subgroup, * p < 0.05)

^b^p values for intergroup comparison (increased LDL-C vs. decreased LDL-C group in the pre treatment values, * p < 0.05)

^c^p values for intergroup comparison (increased LDL-C vs. decreased LDL-C group in the changes from pre to post treatment, * p < 0.05)

## Discussion

### Changes in LDL-C and its subspecies after SGLT-2 inhibitor treatment

Several phase III studies of SGLT-2 inhibitors—with larger sample sizes—have demonstrated that SGLT-2 inhibitors elevate LDL-C levels [5, 6]. Increased LDL-C might increase atherogenic risk in patients treated with SGLT-2 inhibitors. Conversely, the majority of studies have demonstrated that SGLT-2 inhibitors reduce TG and increase HDL-C levels, which reduce atherogenic risk [2, 22]. TG-lowering agents, such as fibrates and omega-3 fatty acids, have a tendency to increase LDL-C [23, 24] probably because of reduced lipid transfer between TG-rich lipoprotein (TRL)-TG and LDL-C [25]. Therefore, it is not surprising that reduced levels of TG associated with SGLT-2 inhibitor use resulted in suppressed generation of cholesterol-poor LDL particles. Another possible mechanism for increases in LDL-C concentrations induced by SGLT-2 inhibitors is an amelioration of insulin resistance by reducing body weight and glucose toxicity [1, 2]. Enhanced insulin sensitivity increases lipoprotein lipase activity, stimulating conversion from very-low-density lipoprotein-C to LDL-C [26]. It is of interest that an insulin sensitizer, pioglitazone, increases LDL-C levels by stimulating LDL-C production—most likely because of enhanced lipoprotein lipase activity [27, 28]. Very recently, Briand et al. [29] reported that empagliflozin reduced LDL receptor-mediated LDL clearance by the liver in hamsters fed atherogenic diets. Taken together, the possible mechanisms for increased LDL-C levels are: (1) increased LDL-C production by enhanced lipoprotein lipase activity, (2) suppressed conversion from cholesterol-rich lb LDL-C to cholesterol-poor sd LDL-C, and (3) impaired LDL-C catabolism by reduced LDL receptors.

We did not observe associated increases in LDL-C levels with the use of dapagliflozin treatment in this study. The individuals whose LDL-C levels were elevated after dapagliflozin treatment had higher TG levels than those whose LDL-C levels were declined, suggesting critical role of TG in SGLT2 inhibitor-induced elevation of LDL-C. In addition, there were significantly differences in sd LDL-C, HDL-C, HDL2-C and lb LDL-C levels between the increased LDL-C and decreased LDL-C subgroups. Higher TG, higher sd LDL-C and lower large sized HDL-C levels are usually observed in the patients who have more visceral obesity [30]. It is known that East Asian type 2 diabetes is characterized by generally lesser obesity and higher insulin sensitivity compared with Caucasians [31]. Therefore, our study is not unusual, and a report from Asian country has also shown that SGLT2 inhibitor unchanged LDL-C concentrations [32].

Despite unchanged levels of LDL-C, dapagliflozin markedly decreased the levels of potent atherogenic sd LDL-C and increased levels of the less atherogenic lb LDL-C. Lb LDL-C levels were further elevated in the subset of patients whose LDL-C levels were increased during dapagliflozin treatment. This would be due to decreased LDL-C catabolism in these patients [29]. Our results imply that lb LDL-C is the sole contributor to rising LDL-C levels in patients using SGLT-2 inhibitors. Even though lb LDL-C is less atherogenic than sd LDL-C, an increase in lb LDL-C could increase cardiovascular risk. However, the Quebec Cardiovascular study revealed that increased lb LDL-C was not positive risk factor but, in fact, a negative risk factor for cardiovascular death [33]. We failed to demonstrate a significant association between changes in TG and changes in LDL-C subspecies during dapagliflozin treatment. However, TG levels fluctuate over the course of a day and postprandial TG levels also influence LDL-C size [34]. Thus, it is possible that dapagliflozin suppresses postprandial TG levels, which affect LDL-C subspecies more strongly than fasting TG levels do. Consistent with previous reports [35, 36], dapagliflozin decreased body weight and liver transaminase levels, and increased adiponectin concentrations. Non-alcoholic fatty liver disease (NAFLD) is the most common cause of elevated liver transaminase levels. NAFLD covers a spectrum, ranging from simple stenosis in the absence of inflammation to non-alcoholic steatohepatitis (NASH). Many large population-based studies have convincingly demonstrated that an elevated serum level of liver transaminase, especially ALT, is a common laboratory surrogate marker for NAFLD and NASH [37]. Patients who have NAFLD or NASH with increased concentrations of sd LDL-C [38, 39] and decreased concentrations of adiponectin [40], carry an additional cardiovascular risk. In this study, average liver transaminase levels were significantly decreased after the administration of dapagliflozin. In addition, AST was reduced in 65%, and ALT was reduced in 73% of patients. This suggests that the protective effects of dapagliflozin on atherogenic fatty liver disease including NAFLD or NASH, occurred concomitantly with type 2 diabetes. We failed to observe a significant correlation between changes in clinical parameters and changes in the LDL-C subspecies. A decrease in sd LDL-C mediated by SGLT-2 inhibitors would be involved in multiple ameliorations of insulin resistance, hypertriglyceridemia, and liver steatosis, which synergistically contribute to suppressed generation of this potent atherogenic lipoprotein.

### Changes in HDL-C and its subspecies after SGLT-2 inhibitor treatment

It is well documented that SGLT-2 inhibitors increase HDL-C levels [2], but so far no study has explored the changes in HDL-C subspecies. The present study has, for the first time, revealed that dapagliflozin specifically increased HDL2-C without affecting HDL3-C. It remains to be proven which HDL2-C or HDL3-C particles are more atheroprotective. Nevertheless, it is known that, while HDL2-C levels are sensitive to and decreased by increases in plasma TG levels [19], adiposity [41], insulin resistance, and are associated with a low risk for incident of type 2 diabetes [42], HDL3-C levels remain fairly constant. Therefore, HDL2-C inversely reflects metabolic burden leading to the development of atherosclerosis. Selective increases in HDL2-C by dapagliflozin may imply ameliorations of hypertriglyceridemia, overweight and insulin resistance, which are also implicated as possible mechanisms for reduced sd LDL-C concentrations.

Recently, a meta-analysis of 21 phase 2b/3 dapagliflozin clinical trials indicated no increased risk for major adverse cardiovascular events with dapagliflozin [43]. Recent report also indicate that dapagliflozin treatment for up to 104 weeks was well tolerated in older patients [44], who are included in a high-risk population for cardiovascular diseases. Additionally, dapagliflozin treatment was associated with reduction of oxidative stress in patients with type 2 diabetes, which may benefit the cardiovascular system [45]. The favourable or neutral effects of dapagliflozin on the cardiovascular diseases risk found in this study may be associated with blood lipid profiles after dapagliflozin treatment.

### Changes in lipid profiles after sitagliptin treatment

We evaluated the effect of a DPP-4 inhibitor, sitagliptin, on plasma lipids and subspecies of LDL-C and HDL-C. Nakamura et al. [46] reported that 25, 50, 100 mg/day of sitagliptin treatment did not change the TG and LDL-C levels. Conversely, the treatment significantly reduced HDL-C levels for 12 months. Our present study showed that sitagliptin exhibited neutral effect on lipids and the subspecies of lipoproteins, which is in keeping with previous reports [47, 48]. Interestingly, Matikainen et al. reported that vildagliptin suppressed postprandial increases in TG [49], and that this suppression was associated with an increase in LDL-C particle diameter [50]. The conflicting result may be in part due to the amount of sitagliptin administrated. We used 50 mg/day of sitagliptin as a standard dose for Japanese patients, while 100 mg/day is the standard dose of sitagliptin in Western countries. Nevertheless, the results obtained from the sitagliptin-treated group may strengthen the validity of the results that dapagliflozin powerfully alters LDL-C and HDL-C subspecies.

### Study limitations

Limitations of this study included the small number of study patients and short treatment period. Therefore, further studies are needed to verify the findings in this study.

## Conclusions

In conclusion, an SGLT-2 inhibitor, dapagliflozin suppressed potent atherogenic sd LDL-C and increased HDL2-C. Although LDL-C was elevated by treatment with dapagliflozin, this increase was solely attributable to elevations in levels of the less atherogenic lb LDL-C. However, these findings were not observed after treatment with DPP-4 inhibitor, sitagliptin. Therefore, the use of new type of glucose-lowering agent, SGLT-2 inhibitor is unlikely to increase atherogenic risk.

## Additional file

**Additional file 1.** Additional tables.

## Abbreviations

SGLT-2: sodium-glucose co-transporter-2
TG: triglyceride
HDL-C: high-density lipoprotein-cholesterol
HDL2-C: high-density lipoprotein 2-cholesterol
HDL3-C: high-density lipoprotein 3-cholesterol
LDL-C: low-density lipoprotein-cholesterol
lb LDL-C: large buoyant low-density lipoprotein-cholesterol
sd LDL-C: small dense low-density lipoprotein-cholesterol
DPP-4: dipeptidyl peptidase-4
RLP-C: remnant-like particle-cholesterol
AST: aspartate aminotransferase
ALT: alanine aminotransferase
apo: apolipoprotein
TRL-TG: triglyceride-rich lipoprotein- triglyceride
NASH: non-alcoholic steatohepatitis
NAFLD: non-alcoholic fatty liver disease
