# Correction to: Dapagliflozin decreases small dense low-density lipoprotein-cholesterol and increases high-density lipoprotein 2-cholesterol in patients with type 2 diabetes: comparison with sitagliptin

**DOI:** 10.1186/s12933-017-0608-5

**Published:** 2017-11-13

**Authors:** Toshiyuki Hayashi, Tomoyasu Fukui, Noriko Nakanishi, Saki Yamamoto, Masako Tomoyasu, Anna Osamura, Makoto Ohara, Takeshi Yamamoto, Yasuki Ito, Tsutomu Hirano

**Affiliations:** 1 Division of Diabetes, Metabolism, and Endocrinology, Department of Medicine, Showa University School of Medicine, 1-5-8, Hatanodai, Shinagawa-ku, Tokyo, 142-8666 Japan; 2 Reagent R&D Department, Denka Seiken Co., Ltd., Nihonbashi Mitsui Tower, 1-1, Nihonbashi-Muromachi 2-chome, Chuo-ku, Tokyo, 103-8338 Japan

## Correction to: Cardiovasc Diabetol (2017) 16:8 DOI 10.1186/s12933-016-0491-5

Following publication of the original article [1], the authors identified a number of errors. In Result (P.3), Table 1 (P.4), Table 5 (P.9) and Supplementary Table 1, the correct unit for adiponectin was μg/mL. In Table 1 (P.4), the correct value for the post treatment body weight in dapagliflozin was 76.2±14.8. In Table 6 (P.10), the correct value for the pre treatment sd LDL/LDL-C in decreased LDL-C group was 0.38±0.10.
