# Supplementary material for: Dapagliflozin decreases small dense low-density lipoprotein-cholesterol and increases high-density lipoprotein 2-cholesterol in patients with type 2 diabetes: comparison with sitagliptin
Source: Cardiovasc Diabetol. 2017 Jan 13;16:8. doi: 10.1186/s12933-016-0491-5 (PMC5237208; doi:10.1186/s12933-016-0491-5)
Supplement: Supplementary file 1 — Additional file 1. Additional tables. [file 12933_2016_491_MOESM1_ESM.docx]

**Supplementary table 1**

**Baseline general characteristics and blood biochemical measurements in dapagliflozin and sitagliptin group**

|  | Dapagliflozin (N=40) | Sitagliptin (N=40) | p^1)^ |
| --- | --- | --- | --- |
| Sex (male/female, n) | 32/8 | 30/10 | 0.444 |
| Age (years) | 53.8±8.3 | 54.2±8.6 | 0.636 |
| Duration of diabetes (years) | 8.2±5.2 | 8.3±5.5 | 0.424 |
| BMI (kg/m^2^) | 28.0±3.8 | 27.9±3.7 | 0.802 |
| SBP (mmHg) | 130.7±15.8 | 133.2±17.8 | 0.632 |
| DBP (mmHg) | 86.9±10.7 | 88.1±9.5 | 0.425 |
| HR (bpm) | 82.5±10.4 | 81.5±10.3 | 0.542 |
| Hb (mg/dL) | 14.4±1.2 | 14.2±1.4 | 0.395 |
| Ht (%) | 41.9±3.5 | 41.4±4.3 | 0.545 |
| AST (IU/L) | 34.5±19.4 | 33.2±9.8 | 0.729 |
| ALT (IU/L) | 46.6±37.0 | 42.8±15.0 | 0.558 |
| γGTP (IU/L) | 53.2±43.0 | 50.9±18.1 | 0.432 |
| BUN (mg/dL) | 14.6±4.3 | 13.5±4.5 | 0.348 |
| Cre (mg/dL) | 0.72±0.17 | 0.77±0.17 | 0.784 |
| eGFR (mL/min/1.73m^2^) | 86.2±18.4 | 83.5±22.7 | 0.616 |
| FPG (mg/dL) | 145.8±47.8 | 144.9±57.9 | 0.426 |
| HbA1c (%) | 7.61±1.15 | 7.55±1.64 | 0.855 |
| C-peptide (ng/mL) | 2.79±1.39 | 2.70±1.25 | 0.515 |
| Adiponectin (ng/mL) | 6.0±3.4 | 6.2±5.3 | 0.602 |
| Antidiabetic medications (%) |  |  |  |
| Metformin | 60 | 65 | 0.581 |
| Sulphonylureas | 38 | 33 | 0.579 |
| α-Glucosidase | 5 | 10 | 0.409 |
| Antihypertensive agents (%) |  |  |  |
| ACEI or ARB | 60 | 53 | 0.416 |
| CCB | 40 | 30 | 0.305 |
| Hypolipidemic agents (%) |  |  |  |
| Statins | 30 | 25 | 0.567 |
| Fibrates | 18 | 13 | 0.499 |

Data are expressed as mean ± standard deviation or number of patients.

^1)^: The unpaired t-test (for continuous variables) or Fisher’s exact test (for categorical variables) was used for statistical analysis of differences in the baseline clinical parameters of the subjects between dapagliflozin and sitagliptin groups.

SBP: systolic blood pressure, DBP: diastolic blood pressure, HR: heart rate, Hb: hemoglobin, Ht: hematocrit,
AST: aspartate aminotransferase, ALT: alanine aminotransferase, γGTP: γ-glutamyltranspeptidase, BUN: blood urea nitrogen, Cre: creatinine, eGFR: estimated glomerular filtration rate, FPG: fasting plasma glucose, ACE: angiotensin-converting enzyme inhibitor, ARB: angiotensin-receptor blocker, CCB: calcium channel blocker

**Supplementary table 2**

**Baseline lipid profiles in dapagliflozin and sitagliptin group**

|  | Dapagliflozin (N=40) | Sitagliptin (N=40) | p^1)^ |
| --- | --- | --- | --- |
| Total-C (mg/dL) | 193.5±36.6 | 192.5±58.2 | 0.867 |
| TG (mg/dL) | 152.6±63.7 | 150.2±132.3 | 0.680 |
| HDL-C (mg/dL) | 48.4±11.1 | 50.3±9.25 | 0.470 |
| LDL-C (mg/dL) | 118.2±32.1 | 120.2±35.1 | 0.447 |
| Non HDL-C (mg/dL) | 145.1±36.0 | 142.2±57.3 | 0.401 |
| LDL-C/HDL-C | 2.54±0.86 | 2.44±0.81 | 0.338 |
| Apo AI (mg/dL) | 133.5±21.6 | 134.9±25.2 | 0.840 |
| Apo AII (mg/dL) | 29.7±4.5 | 30.5±7.2 | 0.590 |
| Apo B (mg/dL) | 100.1±24.5 | 102.3±27.7 | 0.811 |
| Apo CII (mg/dL) | 4.8±1.7 | 5.4±2.8 | 0.702 |
| Apo CIII (mg/dL) | 10.5±3.2 | 10.1±4.5 | 0.618 |
| Apo E (mg/dL) | 4.4±1.2 | 4.3±1.5 | 0.688 |
| Apo AI/apo B | 1.42±0.46 | 1.44±0.37 | 0.780 |
| RLP-C (mg/dL) | 6.8±4.3 | 6.9±8.0 | 0.804 |
| HDL2-C (mg/dL) | 26.1±8.2 | 26.6±7.9 | 0.710 |
| HDL3-C (mg/dL) | 22.2±3.9 | 23.3±5.2 | 0.342 |
| Sd LDL-C (mg/dL) | 54.4±24.6 | 54.0±22.5 | 0.767 |
| Lb LDL-C (mg/dL) | 63.8±27.6 | 66.2±26.3 | 0.917 |
| Sd LDL-C/LDL-C | 0.46±0.16 | 0.43±0.12 | 0.621 |

Data are expressed as mean ± standard deviation.

^1)^: The unpaired t-test was used for statistical analysis of differences in the baseline clinical parameters of the subjects between dapagliflozin and sitagliptin groups.

Total-C: total-cholesterol, TG: Triglycerides, HDL-C: high-density lipoprotein-cholesterol, LDL-C: low-density lipoprotein-cholesterol, Apo: apolipoprotein, RLP-C: remant-like particles-cholesterol, Sd LDL-C: small dense LDL-cholesterol, Lb LDL-C: large buoyant LDL-cholesterol
